# Evaluation of Gel Coating Performance in Extending the Shelf Life of Egg: The Role of Surface Area and Initial Weight

**DOI:** 10.3390/gels10080487

**Published:** 2024-07-23

**Authors:** Thanh Tung Pham, Lien Le Phuong Nguyen, László Baranyai, Mai Sao Dam, Nga Thi Thanh Ha, Adrienn Varga-Tóth, István Dalmadi, Csaba Németh, László Ferenc Friedrich

**Affiliations:** 1 Institute of Food Science and Technology, Hungarian University of Agriculture and Life Sciences, H-1118 Budapest, Hungary; tungpt@hcmute.edu.vn (T.T.P.); nguyen.le.phuong.lien@uni-mate.hu (L.L.P.N.); baranyai.laszlo@uni-mate.hu (L.B.); ngahtt@huit.edu.vn (N.T.T.H.); nemeth.csaba@capriovus.hu (C.N.); friedrich.laszlo.ferenc@uni-mate.hu (L.F.F.); 2 Faculty of Chemical and Food Technology, Ho Chi Minh City University of Technology and Education, Ho Chi Minh 700000, Vietnam; 3 Industrial University of Ho Chi Minh City, Ho Chi Minh 700000, Vietnam; damsaomai@iuh.edu.vn; 4 Faculty of Food Science and Technology, Ho Chi Minh City University of Industry and Trade, Ho Chi Minh 700000, Vietnam

**Keywords:** gel coating, egg size, egg preservation, quality changes, shelf life

## Abstract

This work investigated the impact of chicken egg size, including surface area and initial weight, on the effectiveness of cassava starch-based gel coating during storage at room temperature. The quality of a total of 540 fresh eggs in four different sizes (S, M, L and XL) was evaluated over a 4-week storage period at 25 ± 1 °C (60–65% RH). In this research, images from a scanning electron microscope revealed that the coatings maintained their integrity across all egg sizes, effectively covering pores and cracks throughout storage. The application of gel coating reduced weight loss and preserved the Haugh unit and yolk index, extending freshness by 1–2 weeks compared with uncoated eggs at 25 °C. The results indicated that the performance of the coating varied with egg size. Statistical analysis revealed that the surface area and initial weight of the egg significantly impacted the effectiveness of the coating in preserving quality (*p* < 0.001). Eggs with larger surface areas exhibited a reduced protective effect of the coating, resulting in higher weight loss and lower retention of Haugh unit and yolk index compared with the coated eggs with smaller surface areas. The coating application was more effective in preserving the Haugh unit of eggs with higher initial weights. Overall, the surface area and the initial weight of the egg should be considered as key factors to ensure optimal coating performance.

## 1. Introduction

Chicken eggs are recognized as one of the most valuable and economical animal products. Their rich nutrient content, high digestibility and accessibility have positioned eggs as a fundamental component of the human diet [1]. However, eggs are perishable and susceptible to quality changes during storage due to factors such as moisture evaporation and protein degradation, especially at room temperature [2]. In developing countries, eggs are often sold outdoors due to the dominance of traditional markets. Exposure to high temperatures during storage and transportation can result in significant food waste [3]. Furthermore, European Council Regulation [4] stipulates that eggs should not be refrigerated before reaching the consumer. This leads to the need for alternative methods for maintaining egg quality at sale points.

As a porous material, eggshells allow moisture and carbon dioxide to permeate through their pores. This exchange of gases alters the albumen and yolk, leading to quality degradation [5]. To address this issue, the application of gel coatings to eggs has emerged as a widely recognized, accessible and effective method for maintaining egg quality at room temperature [6,7,8]. Cassava starch, with its low cost, good gas barrier properties and transparency, offers a promising biomaterial for developing such coatings [9]. Additionally, the incorporation of gelatin further enhances the gas barrier of the gel coating by reducing carbon dioxide permeability [6,10]. While previous studies have explored gel coatings for egg preservation, they have primarily focused on incorporating single plasticizers in the gel solutions, using either glycerol or sorbitol [11,12]. However, the use of single plasticizers presents limitations. Glycerol, due to its small molecular size and plasticizing properties, may enhance coating flexibility but can also lead to undesirable softness and adhesion issues. Conversely, sorbitol, with its larger molecular structure and numerous hydroxyl groups, increases tensile strength but may not provide enough flexibility [11,13,14]. This may affect the protective effectiveness of the coating due to the size variations among different egg sizes. By combining the beneficial properties of both plasticizers, the coating ensures consistent protection across all egg shapes and sizes, enhancing its efficacy throughout the storage period. Furthermore, although glycerol may increase water vapor permeability (WVP), the incorporation of sorbitol, a less moisture-absorbent plasticizer, effectively mitigates this effect [11,12]. This approach maintains a lower overall WVP of the coating and provides improved barrier properties. It is important for practical applications in the egg industry, as excessive moisture loss can lead to a decline in overall quality. 

Moreover, it is important to note that many studies have proven the effectiveness of gel coatings in preserving eggs. However, most research has focused on a single-size egg [6,15]. This leaves a significant knowledge gap concerning the efficacy of gel coatings across different egg sizes. Numerous previous studies have revealed that the size and initial weight of an egg affect eggshell characteristics, leading to changes in internal quality at different rates [16,17]. The initial weight of an egg is an important factor that affects both its quality and its grading. Eggs with a higher initial weight typically had a higher yolk-to-albumen ratio, leading to increased lipid oxidation and reduced structural integrity [18]. Furthermore, initial egg weight has been shown to influence eggshell quality, with larger eggs exhibiting a higher density of surface cracks [19]. Consequently, these variations in eggshell quality can influence the preservation performance of the gel coating. Surface area is also a significant factor influencing the rate of egg quality deterioration. Larger eggs, with their greater surface area, experience faster rates of moisture loss and gas exchange compared with smaller eggs. Additionally, they often have thinner shells, increasing their susceptibility to mechanical stress [20,21]. Therefore, the gel coating performance in protecting egg quality will vary across different sizes.

Considering these factors, this study aimed to assess the effectiveness of coating not only on quality parameters like weight loss, Haugh unit and yolk index but also on its performance across different egg surface areas and initial weights. This knowledge is important for developing effective preservation strategies suitable for different sizes of eggs. 

## 2. Results and Discussion

### 2.1. Scanning Electron Microscope

**Figure 1:**
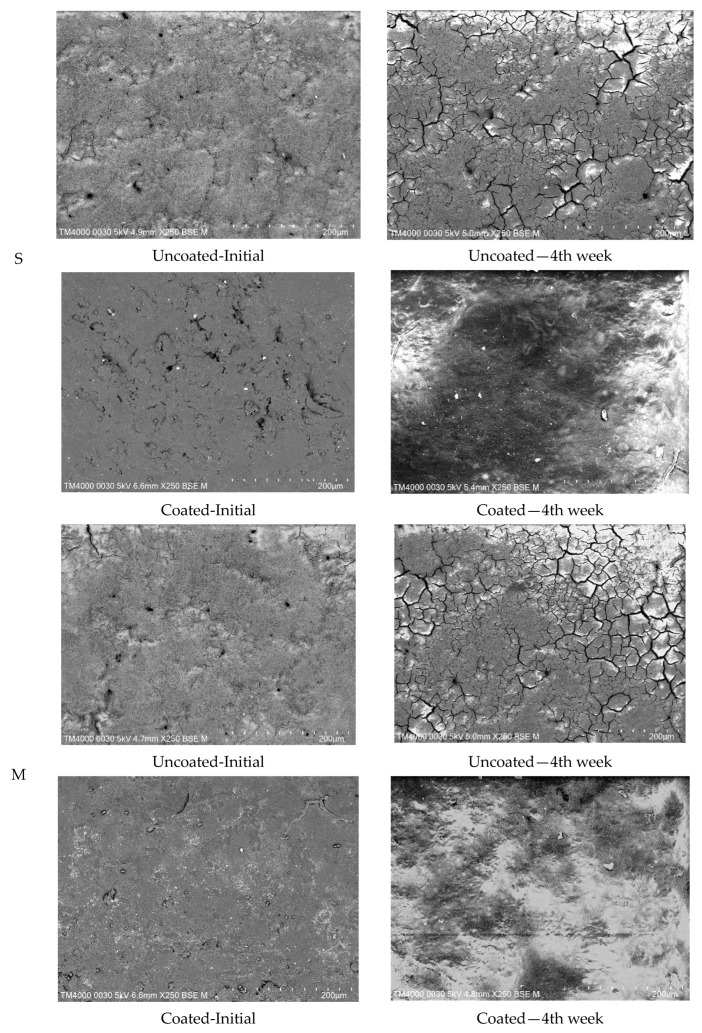

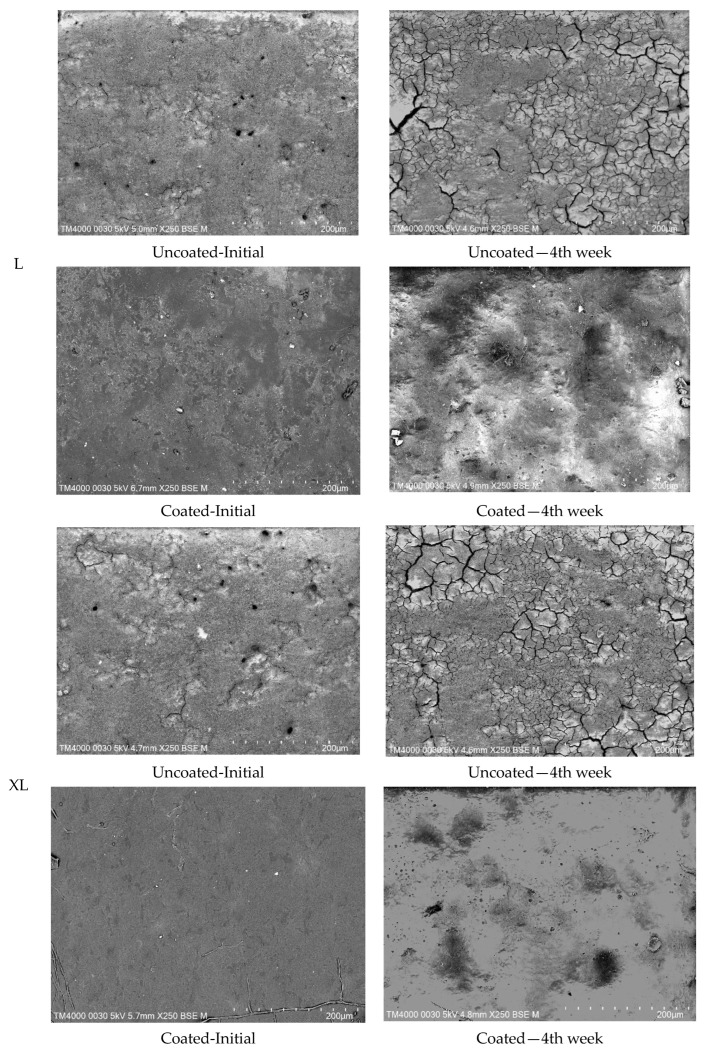
The surface of uncoated and coated eggshells on the initial day and the 28th day.

Figure 1 displays micrographs of the eggshell surfaces for all groups. Initially, the outermost cuticle layer appeared relatively rough and slightly porous, with visible pores. Additionally, some microcracks were evident in the cuticle, likely caused by either membrane drying or handling due to the inherent fragility of eggshells. After 4 weeks of storage, the surface of uncoated eggs showed a significantly greater number of microcracks compared with their initial state. In contrast, no air pores or microcracks were observed on the surface of eggs coated with the gel solution.

### 2.2. Internal Quality of Egg

**Figure 2:**
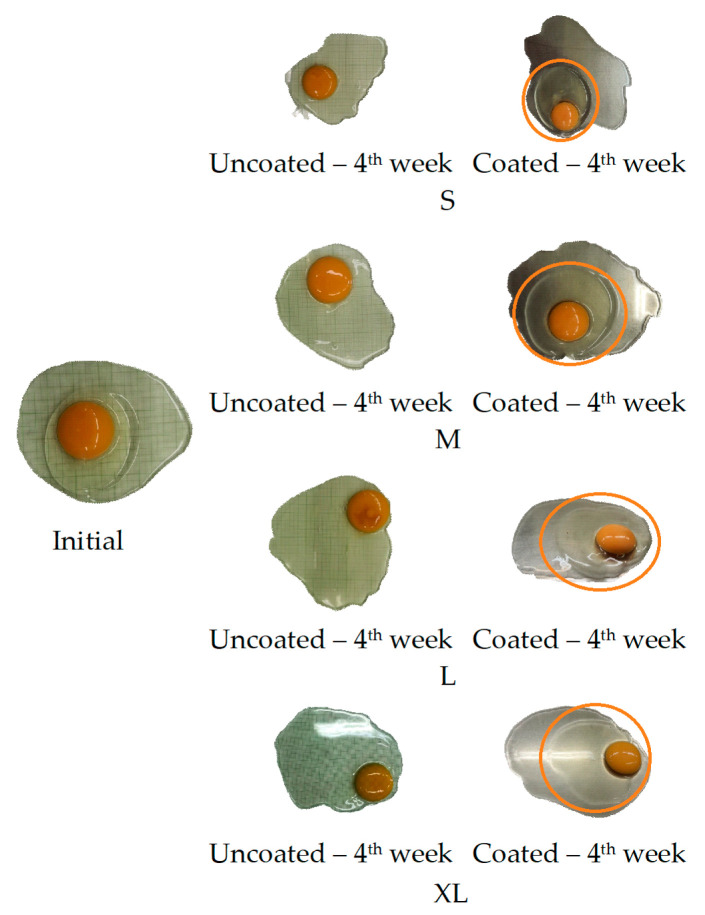
Typical images of eggs during 4 weeks of storage. Orange circle marks the thick albumen.

The data showed that the surface area of chicken eggs differed significantly between size groups (Table 1). On the initial day, eggs obtained a Haugh unit (HU) value ranging from 75.63 ± 1.78 to 92.73 ± 3.22 and a yolk index (YI) ranging from 38.41 ± 0.86 to 47.98 ± 1.98, which classified them as AA and were of excellent quality. However, after 1 week of storage, a significant difference (*p* < 0.05) in internal quality was observed between coated and uncoated eggs. Figure 2 illustrates the visual differences in egg quality observed after 4 weeks of storage at room temperature between the experimental groups. At the end of storage, thick albumen was still observed in the coated eggs. Furthermore, albumen thickness gradually decreased as egg size increased.

**Figure 3:**
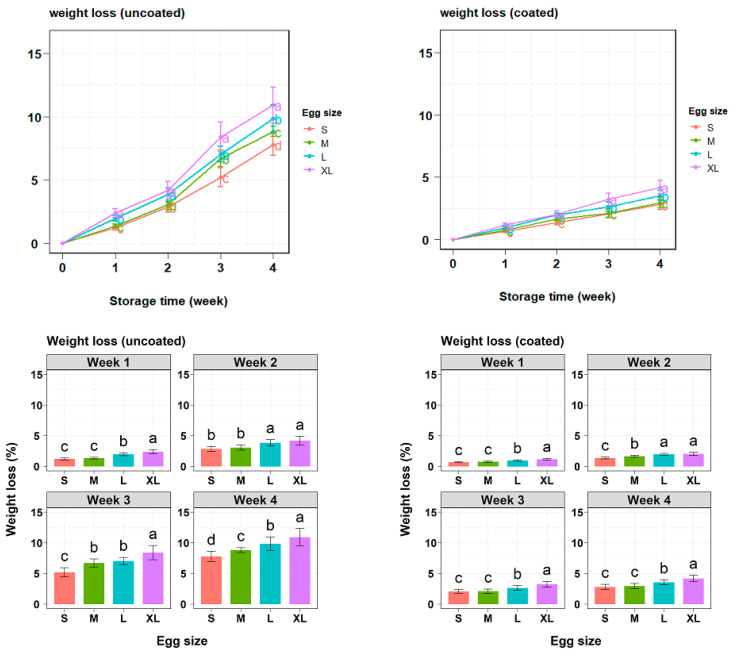
Effect of gel coating on weight loss changes of different egg groups during storage. Lowercase letters are used for size comparisons at the same storage time (Tukey’s, *p* < 0.05).

The weight loss of both coated and uncoated eggs during storage is presented in Figure 3. Generally, the weight loss of eggs increased as storage time was prolonged. However, coated eggs consistently had significantly lower weight loss than uncoated eggs after 7 days of storage. The results showed that the uncoated eggs had varying rates of weight loss in the first week. Size XL eggs suffered the most significant reduction, followed by size L eggs. Size S and M eggs demonstrated the lowest weight loss and were similar to each other. However, after four weeks of storage, clear differences were observed between egg sizes, with larger eggs experiencing greater weight loss compared with the smaller ones. In the coated group, the weight loss rate increased at larger egg sizes in the first week, a trend similar to the uncoated group (Figure 3). After four weeks, statistical analysis showed that there was no significant difference in the rate of weight loss between the smaller egg sizes (S and M) (Table 2). This was in contrast to the uncoated group, where samples of egg size M experienced a greater weight loss rate compared with those of egg size S.

**Figure 4:**
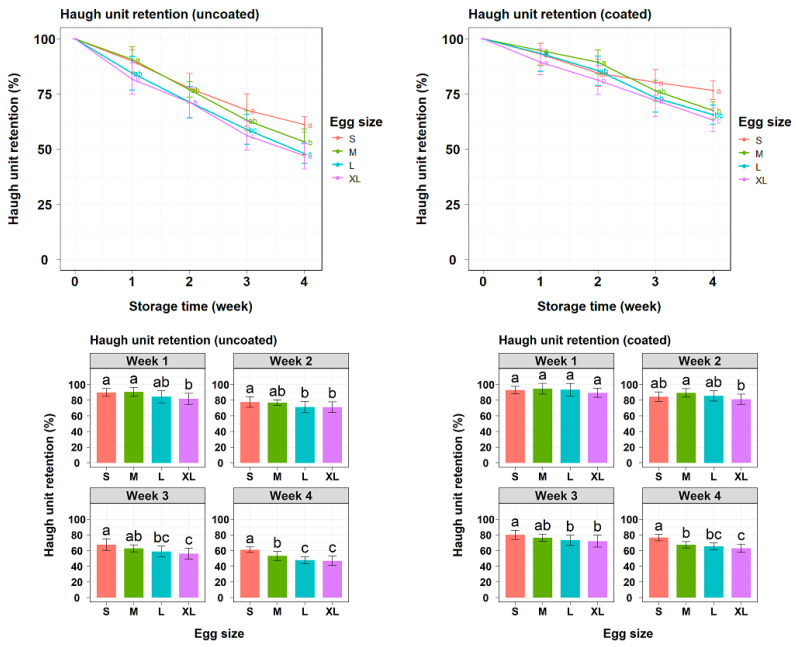
Variation in Haugh unit retention of uncoated and coated eggs over a 4-week period. Lowercase letters are used for size comparisons at the same storage time (Tukey’s, *p* < 0.05).

According to Figure 4, HU retention values generally declined over storage periods, but this decrease was more rapid in uncoated eggs than in gel-coated eggs. Significant differences in HU retention values between the uncoated and coated groups were observed starting from the 7th day of storage. Data indicated an inverse relationship between egg size and HU retention value in uncoated eggs since larger eggs exhibited lower HU retention. However, the application of the gel coating effectively maintained the HU level during the first week, with no significant differences observed between egg sizes. Notably, by the end of the storage period, large-sized eggs, particularly size L, showed improved HU retention compared with size XL eggs (Table 3). This contrasted with uncoated eggs, where no difference in HU retention was observed between sizes L and XL.

**Figure 5:**
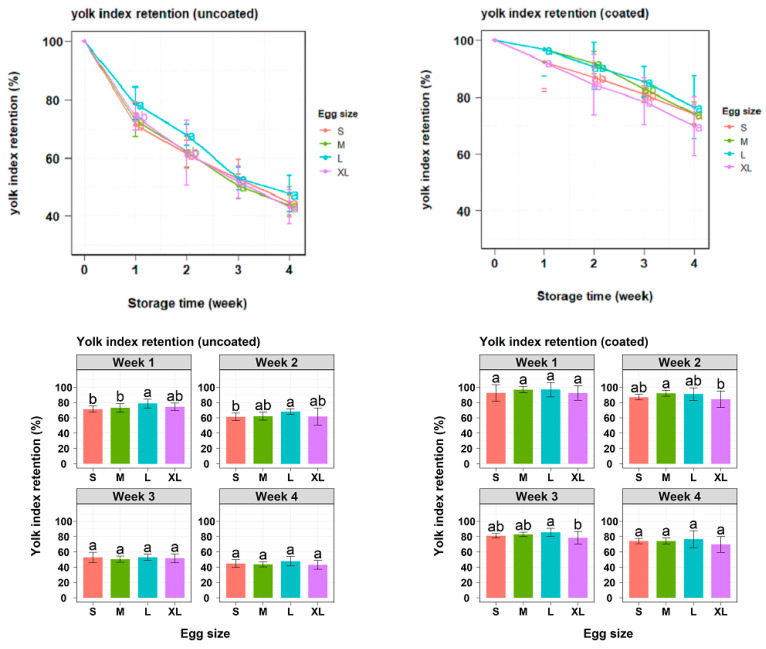
Changes in yolk index retention of uncoated and coated eggs from the initial day to the 4th week. Lowercase letters are used for size comparisons at the same storage time (Tukey’s, *p* < 0.05).

Figure 5 presents the YI retention values recorded during the storage period. Throughout the 4-week study, all eggs exhibited decreasing YI values. The application of gel coating significantly improved the YI retention from the first week of storage, resulting in a smaller change from 18.06% to 23.75% compared with uncoated eggs. By the 28th day of storage, coated eggs presented significantly higher YI retention values (26.67–29.40%) compared with uncoated ones (*p* < 0.05). In general, although egg size influenced the rate of YI decline in uncoated eggs during the first two weeks, this effect was no longer significant in the following two weeks. Conversely, YI retention in coated eggs remained consistent across all egg sizes, with no significant differences observed at the beginning and at the end of the study period (Table 4).

### 2.3. Effect of Egg Surface Area and Initial Weight on Gel Coating Efficiency

Analysis results in Table 5 show a strong fit between the model and measured egg quality parameters, with R^2^ values between 0.84 and 0.94, except for YI retention of coated eggs (R^2^ = 0.65). A low residual standard error and a high F-value provided strong evidence for the overall significance of the model (*p* < 0.001). Further analysis of the general linear model showed that the storage time significantly influenced egg quality in both groups, leading to increased weight loss and a decline in HU and YI retention values over time (*p* < 0.001). In uncoated eggs, the initial weight significantly influenced weight loss but had no impact on HU and YI retention. Conversely, in coated eggs, initial weight significantly affected HU retention but had no effect on weight loss and YI retention. A small but significant influence of surface area was observed on weight loss and HU retention in both groups. Additionally, surface area negatively impacted the YI of coated eggs.

**Figure 6:**
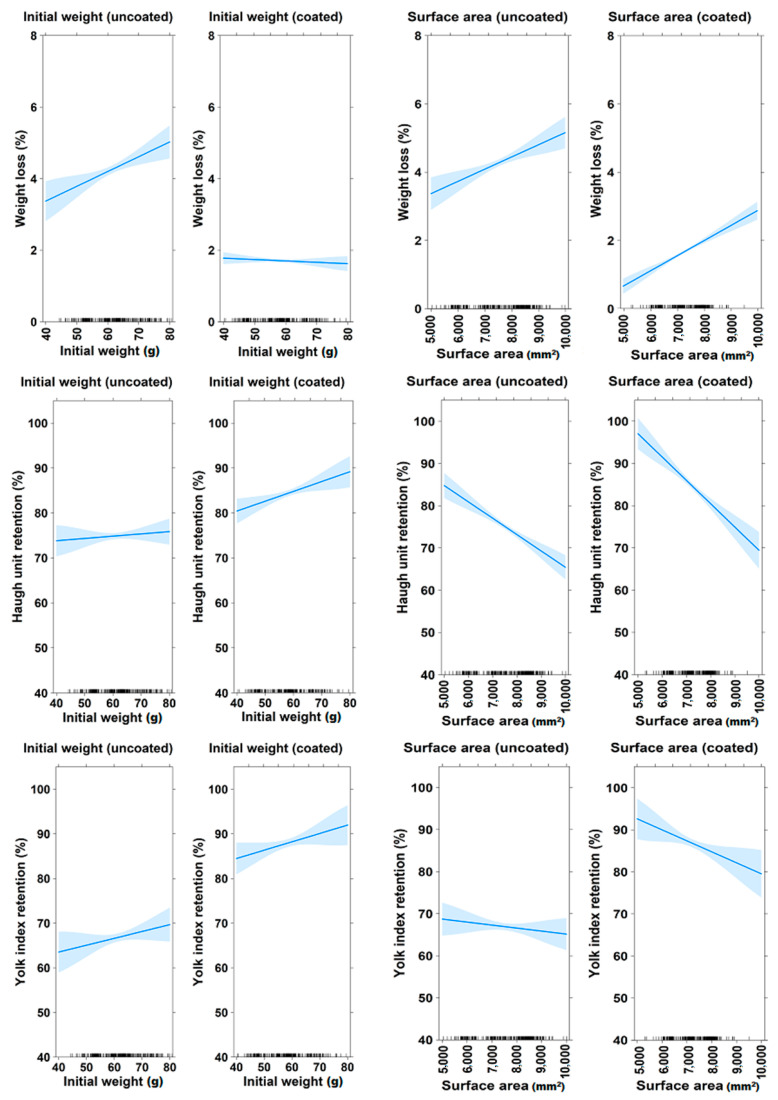
Effect of surface area and initial weight of eggs on weight loss, HU retention and YI retention.

Following the GLM analysis, a plot was created to visualize the influence of surface area and initial egg weight on gel coating efficiency (Figure 6). In general, storage time strongly influenced egg quality in the experiment. However, the rate of quality deterioration of the coated samples was significantly slower than that of the uncoated group. The trend line demonstrates that increasing surface area negatively impacted egg quality parameters, with a corresponding increase in weight loss and a gradual decrease in HU and YI values. Despite this negative impact, coated eggs still maintained significantly higher quality parameters than uncoated eggs with the same surface area. Interestingly, the application of coating proved to be more beneficial for eggs of higher initial weight in terms of HU retention.

### 2.4. Discussion

There is a general consensus that coatings can extend the shelf life of eggs by sealing pores and creating a barrier against external factors [6,8,22]. However, it is important to evaluate whether the coating degrades over time due to constant exposure to environmental conditions and gas exchange. Xu [23] reported that the protective ability of chitosan coating on an egg reduced after 16 days due to the degradation of its dense structure. In contrast, in this study, the gel coating maintained its integrity even after 4 weeks, with no observed air pores or microcracks. Additionally, the coating layers remained intact across all egg sizes. This suggested that cassava starch gel coating, combined with gelatin and plasticizers, possessed improved mechanical properties, successfully maintaining its barrier properties throughout the storage period.

Furthermore, the effect of storage time, initial weight and surface area on egg quality parameters (weight loss, HU and YI) are also evaluated in Table 5. As expected, storage time significantly impacted egg quality in both coated and uncoated groups, causing a general decline over time. However, the application of coating was effective in mitigating this decline and helped maintain egg quality. This observation could be attributed to the dense molecular network formed by the semi-crystalline structure of the cassava starch, which acted as a barrier against gas exchange and moisture loss. The addition of gelatin further strengthened this barrier by forming hydrogen bonds, making the network more cohesive [8,24]. This combined effect created an effective gas barrier, a crucial factor in preserving egg quality during storage.

In egg farms and the food industry, weight loss is an important indicator of egg quality. The reduction in weight is linked to the evaporation of H_2_O and CO_2_ from the albumen through the porous shells, a process that accelerates at room temperature [25]. The FAO [26] suggests that the loss in egg weight from 2% to 3% during storage is acceptable for sale. Previous studies by Homsaard et al. [15] and Oliveira et al. [27] reported weight losses of 4–6% in eggs coated with cassava starch-based solutions after 4 weeks. In this study, the gel coating was more effective, maintaining weight loss below 3% for smaller egg sizes (S and M) for up to four weeks. This enhanced preservation could be attributed to the incorporation of a dual-plasticizer network in the gel coating. Glycerol improved the mechanical properties of the coating, while sorbitol enhanced its barrier properties. This combined effect contributed to a more effective preservation of egg quality [8,24]. In this research, uncoated eggs generally exceeded the acceptable weight loss threshold of 3% after the first week of storage, with weight loss ranging from 3.08% to 4.16% for sizes M to XL. Size S eggs were the exception, maintaining acceptable weight loss until the second week (Figure 3). In contrast, coated eggs maintained acceptable weight loss for up to three weeks for sizes L and XL and up to four weeks for sizes S and M. The ANOVA analysis results in Table 5 show that the initial weight and surface area of eggs correlated with weight loss. This finding is consistent with the perishable nature of eggs. Since eggs are primarily composed of water, a higher initial weight typically corresponds to higher water content [28]. This resulted in a greater concentration gradient, increasing the driving force for moisture loss [29]. A larger surface area could result in a thinner and more porous shell, leading to reduced eggshell strength and accelerated moisture loss [20,30]. For the coated egg, the effect of initial weight on weight loss was not significant (Table 5). This could be attributed to the application of the gel coating altering the water vapor pressure gradient between the interior and exterior environments of the eggs [8,29]. Therefore, initial weight did not influence the effectiveness of coating in reducing weight loss. However, increasing the surface area negatively impacted coating efficiency. As previously discussed, a larger surface area can lead to increased gas release. Although the coating permitted limited gas exchange, a disadvantage of the dipping method was the non-uniformity of coating thickness, resulting in variable protection across the egg surface [8]. This heterogeneity could be exacerbated by increased surface area. However, the application of coating significantly reduced the overall influence of surface area on weight loss.

Furthermore, HU is also one of the important parameters for assessing and describing the quality of eggs. The decrease in HU value indicates the breakdown of the thick gelatinous structure of albumen, leading to a reduction in its height [22]. Bhale et al. [31] and Caner [32] found that chitosan-based coatings maintained HU values around 50 after 4 weeks at 25 °C. In contrast, the gel coating in this study preserved “A” quality (HU > 54) for up to 4 weeks of storage, with the smallest size (S) even maintaining an HU of 71.08. This difference could be due to the combined effect of the ingredients in the coating formula. According to Figure 4, a significantly higher HU retention was observed in the coated group compared with the uncoated group. In addition to the improved barrier properties provided by the combination of cassava starch and gelatin, the mechanical properties of the coating were further enhanced by the addition of plasticizers. By inserting molecules between polymer chains and disrupting intermolecular forces, plasticizers increase the flexibility of the polymer network [33]. This leads to improved adhesion to the egg surface and coating integrity [11,34]. Therefore, the protective effect of larger-sized eggs was also enhanced. The final week ranking results for the coated egg group showed that size L eggs improved in HU retention, close to the level of size M eggs (Table 3). This contrasts with the uncoated large eggs, which exhibited the lowest HU retention capacity (Figure 4). The analysis results indicated that both initial weight and surface area affected the HU retention ability of both uncoated and coated eggs (Table 5). Interestingly, further analysis revealed that while the initial weight was a significant variable in the model, its regression coefficient was not significant within the uncoated egg group (*p* > 0.05). However, in the coated group, higher initial weight positively affected the coating efficiency (*p* < 0.05). This difference might be attributed to the greater influence of the eggshell structure, such as cracks, thickness and air pores, on albumen structure compared with the effect of initial weight in uncoated eggs [20,21,35]. The application of coating mitigated the impact of eggshell properties on egg quality, thereby making the influence of initial weight on the HU retention more apparent. Furthermore, the protective effect of the coating was more pronounced in eggs with a higher initial weight. However, increased surface area negatively impacted the protective effect of coating on HU retention, similar to its effect on weight loss. The results showed that the gas barrier ability of the coating was influenced by the surface area it covers.

The YI value, an industry standard indicator of egg freshness, represents the strength of the vitelline membrane and the spherical shape of the yolk [8]. In this study, the developed gel coating formed an efficient and durable sealing structure on the eggshell, as proved by the significantly higher YI value in coated eggs compared with uncoated ones after storage at 25 °C (Figure 5). Further analysis results showed that the initial weight did not affect the YI retention of both experimental groups (*p* > 0.05). This could happen because the yolk quality mainly depends on the composition and durability of the vitelline membrane and not on the initial egg weight [36]. Notably, the surface area did not influence YI retention in uncoated eggs, while it had a small but significant effect on the coated samples. These observed results might be due to the coating inhibiting gas release and water diffusion from the egg albumen to the yolk, thereby slowing the weakening of the yolk membrane [36]. Therefore, increased surface area was associated with a decrease in the efficiency of coating in maintaining YI. 

These results are visually supported by Figure 6. The chart illustrates the natural decline of egg quality during storage. However, the application of gel coating demonstrated a clear ability to preserve egg quality compared with uncoated eggs. As discussed earlier, surface area and initial weight impacted the efficiency of gel coating. Figure 6 shows that coated eggs with larger surface areas experienced nearly twice the weight loss compared with smaller-sized ones. However, in the case of uncoated eggs, the weight loss was significantly higher. The graph also indicates that increasing egg surface area reduced the HU retention value. Interestingly, for XL-size eggs, the reduction rate in HU retention was similar regardless of whether they were coated or uncoated. In uncoated eggs, YI retention remained nearly constant regardless of the surface area, as shown by the nearly horizontal line. For coated eggs, YI retention decreased as surface area increased, but the overall decline was less pronounced compared with uncoated eggs. Notably, the protective ability of the coating was more effective for eggs with higher initial weight. These visualizations supported the statistical findings, confirming the negative influence of surface area and the positive influence of initial weight on the effectiveness of gel coating.

## 3. Conclusions

This study demonstrated that coated eggs maintained quality and extended shelf life by up to 14 days compared with uncoated eggs at 25 ± 1 °C (60–65% RH). The developed gel coating in this study has shown promising results in prolonging egg freshness and maintaining quality during storage, providing a simple and effective solution for the egg industry. Regarding coating performance, the gel coating demonstrated the greatest efficacy for sizes S and M, with effectiveness gradually decreasing for sizes L and XL, indicating a greater benefit for smaller surface areas. However, within each size category, the coating was observed to be more effective in maintaining the quality of eggs with higher initial weights. This suggests that the protective capacity of the coating is influenced not only by egg surface area but also by other factors, such as the variations in egg composition, which may be more prevalent in heavier eggs. The rate of quality change in relation to surface area and initial weight in this study can serve as a reference to understand how the coating behaves on eggs of different sizes. One point to note is that the egg storage environment can fluctuate significantly throughout the supply chain, from the farm to the retailer. It is important for future studies to evaluate the gel coating performance on different egg sizes while considering additional factors, such as fluctuations in temperature and humidity.

## 4. Materials and Methods

### 4.1. Materials

A total amount of 540 freshly laid, unwashed, brown-shelled eggs without any physical damage were obtained from the farm at Capriovus Ltd. (Szigetcsép, Hungary). Cassava starch powder, gelatin, sorbitol and glycerol, all with a purity of ≥99%, were purchased from various suppliers in Budapest, Hungary: Hunorganic Ltd., Szilasfood Ltd., Parma Produkt Ltd., and Budai Szent Klara Pharmacy, respectively.

**Figure 7:**
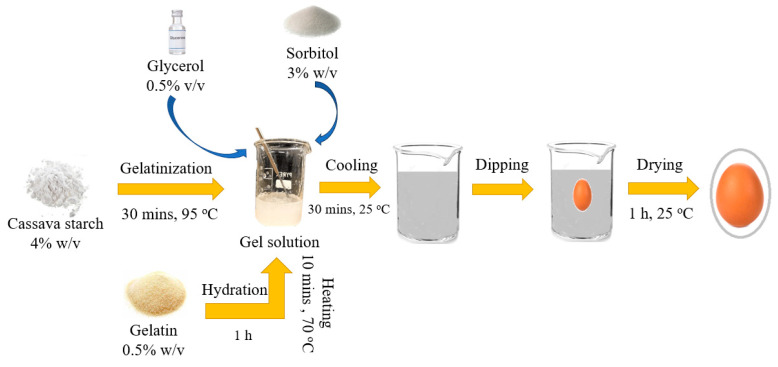
The process of gel coating preparation.

The gel coating preparation is detailed in Figure 7. For the experiment, the eggs were first washed with tap water and then dried with a towel. The eggs were then sorted into four groups, S, M, L and XL, according to the EU egg grading standards [4], with each group containing 135 eggs. A subset of 60 eggs in each group was randomly selected for coating treatment. Those eggs were immersed into the gel coating solution for 15 s and allowed to dry at room temperature for one hour. During the experiment, the eggs were stored in molded fiber containers for up to four weeks.

### 4.2. Eggshell Morphology

The surface of the eggshells was analyzed using a scanning electron microscope (SEM; JEOL Ltd., Tokyo, Japan) at 5 kV and a magnification of 250×. Images of eggshells from both uncoated and coated samples were captured initially upon collection and again after 4 weeks of storage. Three eggs from each treatment group were randomly selected to be photographed each time.

### 4.3. Egg Quality Measurement

Fifteen fresh eggs from each group were randomly selected, and their quality parameters were assessed immediately after collection. Additionally, weekly analyses were conducted, including measurements of weight loss, Haugh unit and yolk index. Weekly measurements used 15 pieces from each group.

Eggs were individually weighed using a digital balance (±0.01 g) (Sartorius AY612, Sartorius^®^, Goettingen, Germany). The percentage of weight loss during storage was calculated weekly based on the initial weight of the egg at the start of the experiment according to the following equation [37]:

$$Weight\;loss\left(\%\right)=\frac{\left(W_{i}-W_{f}\right)}{W_{i}}\times 100,$$

where $W_{i}$ is the initial egg weight (g) or the egg weight (g) after coating for the treatment group, and $W_{f}$ is the current egg weight (g) at the time of measurement.

The albumen height was determined using a digital caliper (Mitutoyo 500-150-30, AOS Absolute Digimatic, Japan), with measurements specifically taken at a 10 mm distance from the yolk. Following this, the Haugh Unit (HU) was calculated using the equation developed by Haugh [38]:

$$HU=100\times \log\left(H-1.7\times W^{0.37}+7.6\right),$$

where H presents the measured albumen height (mm), and W is the egg weight (g).

The HU retention was calculated using this formula:

$$HU\;retention\left(\%\right)=100\times (\frac{{HU}_{m}}{\bar{HU}}),$$

where $\bar{HU}$ is the average HU value of 15 eggs at the initial storage time, and ${HU}_{m}$ is the HU value of an individual egg at the measurement time.

Measurements of both the width and height of the yolk were performed using a digital caliper to determine the yolk index (YI) according to the equation provided by Sharp and Powell [39]:

$$YI=\frac{Yolk\;height}{Yolk\;width}\times 100$$

The YI retention was calculated using this formula:

$$YI\;retention\left(\%\right)=100\times \left(\frac{{YI}_{m}}{\bar{YI}}\right),$$

where $\bar{YI}$ is the average YI value of 15 eggs at the initial storage time, and ${YI}_{m}$ is the YI value of an individual egg at the measured time.

**Figure 8:**
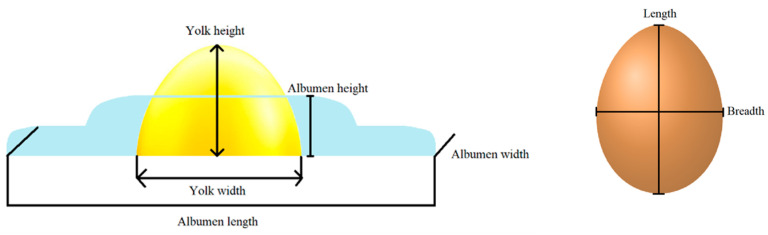
The parameters of egg measurement.

The length (L) and breadth (B) of the eggs were measured with the same digital caliper as in the case of HU (Figure 8). The surface area of the eggs was estimated on the basis of these parameters using the formula provided by Narushin [40]:

$$S=\left(3.155-0.0136L+0.0115B\right)LB$$

### 4.4. Data Analysis

The results were analyzed using a two-way analysis of variance (ANOVA) with SPSS software (version 28.0, SPSS Inc., Chicago, IL, USA). Pairwise comparisons were conducted using Tukey’s post hoc test. The General Linear Model (GLM) was used to assess the effect of egg surface area and egg weight on coating efficiency. Data were presented in the figures as mean ± standard deviation. The significance level of *p* < 0.05 was used throughout the study.

## Figures and Tables

**Table 1:** The surface area of chicken eggs in the experiment. Lowercase letters are used for size comparisons (Tukey’s, *p* < 0.05).

Size	S	M	L	XL
Area (mm^2^)	6603.11 ± 190.71 ^c^	7125.38 ± 163.30 ^b^	7873.93 ± 206.00 ^ab^	8054.96 ± 322.27 ^a^

**Table 2:** The ANOVA results of weight loss (%) of coated eggs over a 4-week period.

	Week	Week 1	Week 2	Week 3	Week 4
Size					
S		0.69 ± 0.09	1.36 ± 0.15	2.08 ± 0.32	2.81 ± 0.42
M		0.77 ± 0.12	1.66 ± 0.16	2.11 ± 0.34	2.96 ± 0.41
L		0.97 ± 0.11	1.97 ± 0.16	2.64 ± 0.39	3.54 ± 0.40
XL		1.17 ± 0.14	2.05 ± 0.27	3.23 ± 0.48	4.19 ± 0.53
*p*-value		1.81 × 10^−16^	5.58 × 10^−14^	1.76 × 10^−11^	1.14 × 10^−11^
CV (%)		12.83808	10.93666	15.42699	13.18279

**Table 3:** The ANOVA results of Haugh unit retention (%) of coated eggs over a 4-week period.

	Week	Week 1	Week 2	Week 3	Week 4
Size					
S		93.07 ± 4.70	84.47 ± 6.12	80.20 ± 5.85	76.65 ± 4.18
M		94.69 ± 6.87	89.40 a ± 5.40	76.41 ± 4.73	67.52 ± 4.03
L		89.45 ± 5.80	81.24 ± 6.56	72.16 ± 7.63	63.10 ± 5.29
XL		93.32 ± 8.11	85.49 ± 6.62	73.20 ± 6.56	65.53 ± 4.28
*p*-value		0.161	0.007	0.004	<0.001
CV (%)		7.01	7.28	8.32	6.55

**Table 4:** The ANOVA results of yolk index retention (%) of coated eggs over a 4-weeks period.

	Week	Week 1	Week 2	Week 3	Week 4
Size					
S		92.57 ± 9.35	86.93 ± 8.21	81.06 ± 6.51	74.13 ± 10.92
M		96.80 ± 4.26	92.09 ± 3.87	82.87 ± 2.91	74.24 ± 4.05
L		96.83 ± 9.43	90.97 ± 8.29	85.52 ± 5.39	76.61 ± 11.07
XL		92.47 ± 9.58	84.32 ± 10.71	78.51 ± 8.24	69.76 ± 10.38
*p*-value		0.326522	0.017156	0.005939	0.142125
CV (%)		9.352369	8.200594	6.508916	10.9207

**Table 5:** The summary of GLM analysis result of the uncoated and coated egg.

**Weight loss (%)**				
	Uncoated		Coated	
	F value	Pr (>F)	F value	Pr (>F)
Storage time	4595.22	<2 × 10^−16^ ***	4157.58	<2 × 10^−16^ ***
Initial weight	182.88	<2 × 10^−16^ ***	220.35	<2 × 10^−16^ ***
Surface area	14.13	0.000205 ***	79.04	<2 × 10^−16^ ***
R^2^	0.9418		0.9377	
Residual standard error	0.8592		0.3179	
GLM model coefficients for weight loss				
	t value	Pr (>|t|)	t value	Pr (>|t|)
(Intercept)	−14.011	<2 × 10^−16^ ***	−16.821	<2 × 10^−16^ ***
Storage time	68.059	<2 × 10^−16^ ***	63.955	<2 × 10^−16^ ***
Initial weight	3.433	0.000681 ***	−0.528	0.598
Surface area	3.759	0.000205 ***	8.89	<2 × 10^−16^ ***
**Haugh unit retention (%)**				
	Uncoated		Coated	
	F value	Pr (>F)	F value	Pr (>F)
Storage time	3024.38	<2 × 10^−16^ ***	1490.13	<2 × 10^−16^ ***
Initial weight	110.08	<2 × 10^−16^ ***	45.85	6.85 × 10^−11^ ***
Surface area	45.16	9.31 × 10^−11^ ***	51.48	5.83 × 10^−12^ ***
R^2^	0.9148		0.8428	
Residual standard error	5.369		5.088	
GLM model coefficients for Haugh unit retention				
	t value	Pr (>|t|)	t value	Pr (>|t|)
(Intercept)	44.92	<2 × 10^−16^ ***	44.411	<2 × 10^−16^ ***
Storage time	−54.975	<2 × 10^−16^ ***	−38.134	<2 × 10^−16^ ***
Initial weight	0.641	0.522	2.965	0.00328 **
Surface area	−6.72	9.31 × 10^−11^ ***	−7.175	5.83 × 10^−12^ ***
**Yolk index retention (%)**				
	Uncoated		Coated	
	F value	Pr (>F)	F value	Pr (>F)
Storage time	2094.988	<2 × 10^−16^ ***	538.646	<2 × 10^−16^ ***
Initial weight	1.705	0.193	0.381	0.538
Surface area	0.737	0.391	6.113	0.014 *
R^2^	0.8763		0.6481	
Residual standard error	7.103		6.893	
GLM model coefficients for Yolk index retention				
	t value	Pr (>|t|)	t value	Pr (>|t|)
(Intercept)	22.728	<2 × 10^−16^ ***	27.824	<2 × 10^−16^ ***
Storage time	−45.515	<2 × 10^−16^ ***	−22.999	<2 × 10^−16^ ***
Initial weight	1.392	0.165	1.858	0.0641
Surface area	−0.858	0.391	−2.473	0.014 *

* *p* < 0.05, ** *p* < 0.01, *** *p* < 0.001.

## Data Availability

The data presented in this study are available on request from the corresponding author.
